# Phosphorus and naphthalene acetic acid increased the seed yield by regulating carbon and nitrogen assimilation of flax

**DOI:** 10.3389/fpls.2023.1228755

**Published:** 2023-08-30

**Authors:** Yaping Xie, Huirong Duan, Limin Wang, Jianping Zhang, Kongjun Dong, Xingrong Wang, Yanjun Zhang, Yangchen Zhou, Wenjuan Li, Yanni Qi, Wei Zhao, Zhao Dang, Xingzhen Wang, Wen Li, Lirong Zhao

**Affiliations:** ^1^ Crop Research Institute, Gansu Academy of Agricultural Sciences, Lanzhou, China; ^2^ College of Agronomy, Gansu Agricultural University, Lanzhou, China; ^3^ Lanzhou Institute of Husbandry and Pharmaceutical Science, Chinese Academy of Agricultural Sciences, Lanzhou, China

**Keywords:** flax, phosphorus, naphthylacetic acid, carbon and nitrogen assimilation, seed yield

## Abstract

To evaluate the impact of phosphorus (P) combined with exogenous NAA on flax yield, enhance flax P utilization efficiency and productivity, minimize resource inputs and mitigate negative environmental and human effects. Therefore, it is crucial to comprehend the physiological and biochemical responses of flax to P and naphthylacetic acid (NAA) in order to guide future agronomic management strategies for increasing seed yield. A randomized complete block design trial was conducted under semi-arid conditions in Northwest China, using a factorial split-plot to investigate the effects of three P (0, 67.5, and 135.0 kg P_2_O_5_ ha^–1^) and three exogenous spray NAA levels (0, 20, and 40 mg NAA L^–1^) on sucrose phosphate synthase (SPS) and diphosphoribulose carboxylase (Rubisco) activities as well as nitrogen (N) and P accumulation and translocation in flax. Results indicated that the SPS and Rubisco activities, N and P accumulation at flowering and maturity along with assimilation and translocation post-flowering, fruiting branches per plant, tillers per plant, capsules per plant, and seed yield were 95, 105, 14, 27, 55, 15, 13, 110, 103, 82, 16, 61, 8, and 13% greater in the P treatments compared to those in the zero P treatment, respectively. Moreover, those characteristics were observed to be greater with exogenous spray NAA treatments compared to that no spray NAA treatment. Additionally, the maximum SPS and Rubisco activities, N and P accumulation, assimilation post-flowering and translocation, capsules per plant, and seed yield were achieved with the application of 67.5 kg P_2_O_5_ ha^–1^ with 20 mg NAA L^–1^. Therefore, these findings demonstrate that the appropriate combination of P fertilizer and spray NAA is an effective agronomic management strategy for regulating carbon and nitrogen assimilation by maintaining photosynthetic efficiency in plants to increase flax productivity.

## Introduction

Flax (*Linum usitatissimum* L.), a C_3_ plant species, exhibits versatility as a valuable source for food, industry, and bioenergy (Zuk et al., 2015; Parikh et al., 2019; Xie et al., 2020). With the global human population projected to continue growing in the coming decades, current rates of crop productivity may not be sufficient to meet future demand for food (Long et al., 2015). Additionally, accelerating global warming poses a threat to crop production. Hence, improving crop productivity under future climatic conditions is a huge challenge (Suganami et al., 2021). One promising strategy for achieving this goal is to enhance carbon (C) and nitrogen (N) assimilation (Ren et al., 2020) and photosynthetic efficiency (Makino, 2011; Ort et al., 2015).

Phosphorus (P), as the second macronutrient for plants, plays a crucial role in various metabolic activities (Singh et al., 2018; Yaakob et al., 2021). Numerous studies have focused on the effect of P on photosynthesis (Singh et al., 2018; Taliman et al., 2019; Li et al., 2022; Chai et al., 2023; Kayoumu et al., 2023). Photosynthesis is primarily driven by sucrose, which serves as the main photoassimilate transported from source to sink tissues and storage in higher plants (Okamura et al., 2011; Chen et al., 2012). Previous literature has documented that sucrose phosphate synthase (SPS), a pivotal rate-limiting enzyme during the sucrose biosynthesis process in plants (Liao et al., 2022), displays differential expression patterns (Wang et al., 2018). Moreover, ribulose-1,5-bisphosphate carboxylase/oxygenase (Rubisco) is considered a crucial targets for enhancing photosynthetic capacity (Parry et al., 2013; Carmo-Silva et al., 2015; Sharwood, 2017) and determining the rates of CO_2_ assimilation in C_3_ leaves commonly (Lawlor, 2002). Nitrogen is below required to sustain the protein synthesis, which is inadequate for maximum CO_2_ assimilation (Lawlor et al., 1989). Recently, the expression of *SPS* gene families has been studied in various plant species, including rice (*Oryza sativ*a L) (Okamura et al., 2011; Mulyatama et al., 2022), litchi (*Litchi chinensis* Sonn) (Wang et al., 2018), cassava (*Manihot esculenta Crantz*) (Huang et al., 2020), and kiwi fruit (*Actinidia chinensis Planch*) (Liao et al., 2022). Studies on *Rubisco* genes have also been performed in rice by Suzuki et al. (2017). However, the relative expression levels of the SPS gene family and Rubisco gene in flax have not been reported. Furthermore, research has shown that P plays a significant role in regulating N uptake (Güsewell, 2004), N and P accumulation (Dordas, 2009) and translocation (Güsewell, 2004; Dordas, 2009). Additionally, numerous studies have demonstrated that an appropriate quantity P can significantly enhance the seed yield of oilseed crops such as flax (Xie et al., 2020; Xie et al., 2022), soybean (*Glycine max*) (Yin et al., 2016; Taliman et al., 2019), canola (*Brassica napus* L.) (Gao and Ma, 2015), crambe (*Crambe abssynica* Hoechst) (Rogério et al., 2013), and sunflower (*Helianthus annuus* L.) (Abbadi and Gerendás, 2011). Nevertheless, excessive application of P in agriculture not only leads to poor yield and increases production cost, but also causes severe environmental problems. Therefore, optimizing the management of P fertilization is important for maximizing flax productivity with minimal energy inputs and negative environment effects in flax production.

Auxin is a crucial plant growth promoter (Giannakoula et al., 2012) that plays a vital role in various respects, including flowering, fruiting, and seed formation (Giannakoula et al., 2012). The use of auxin has opened up new possibilities for increasing seed production in legume cultivation (Zhang et al., 2009). According to Lorenzetti (1993), the use of synthetic growth regulators can achieve seed yields in grasses similar to those obtained through genetic manipulation in wheat (*Triticum aestivum* L.), rice (*Oryza sativa* L.), and barley (*Hordeum vulgare* L.) by breeders. Study on the effect of growth hormones on foliage has largely focused on applications near the flowering stage due to the auxin’s crucial role in seed development (Mousavi et al., 2022). In oil crops, there have been reports demonstrated the positive effect of auxin on seed yield and yield components of flax (Rastogi et al., 2013) and safflower (*Carthamus tinctorius* L.) (Mousavi et al., 2022). In addition, previous research (Giannakoula et al., 2012) has also suggested that the application of indole-3-acetic acid (IAA) can improve the seed yield of lentil (*Lens culinaris*). Only a limited number of research papers have been published on the impact of plant growth promoters on the mineral nutrition of specific crops. In a study of wheat, Prasad et al. (1991) reported that the application of small amount of plant growth promoter can aid in nutrient absorption, resulting in increased yields. Evidence has demonstrated that NAA positively regulates P translocation within plants and accumulation in wheat grains (Peng et al., 2007). Very little is known about the effect of P and NAA on N and P accumulation and translocation within plants. In agriculture, farmers occasionally apply NAA together with P fertilizers to increase crop yields. Hence, more information is needed on how P and exogenous spray NAA can affect SPS and Rubisco activities and how it can affect N and P assimilation and translocation within flax plants.

The objective of our study was to investigate the effect of P and auxin on the relative expression level of *LuSPSs* gene family and R*ubisco* gene using real-time quantitative PCR (RT-qPCR) techniques, as well as SPS and Rubisco activities in flax leaves. Additionally, we examined N and P accumulation at flowering and maturity, post-flowering N and P assimilation, N and P translocation, N harvest index (NHI) and P harvest index (PHI), and seed yield in flax. Based on the results presented here, we aimed to investigate the effects of different levels of P and NAA on flax productivity, with a view to improving flax’s use of P and yield with fewer inputs of fertilizer while reducing concerns for environmental, ecological, and human health.

## Materials and methods

### Site description, experimental design and treatments

The experiment was conducted at Oil Research Institute, Dingxi Academy of Agricultural Science, Gansu Province, China (35°48′ N, 104°49′ E, altitude of 2050 m) in both 2019 and 2020. The experimental site has a continental climate. The soil type is Arenosols (FAO, 2015), with wheat as the previous crop. During the growing season from March to August, monthly temperatures ranged from −7 to 32°C in Dingxi, with the lowest temperature recorded in March and the highest value in July. The mean monthly temperatures each year were close to the long-term average (30 yr). In summary, the total precipitation during the growing season from March to August was between 309 and 317 mm in both 2019 and 2020.

The experiment was designed as a split-plot randomized complete block design with three replicates. The plot size was 5.0m×4.0m. Flax cultivar Lunxuan 2 was sown on 2 and 9 April in 2019 and 2020, at a seeding rate of 1050 viable seeds m^–2^ for targeting 750 plants m^–2^. The main plots were assigned three P rates (0, 67.5, and 135.0 kg P_2_O_5_ ha^–1^), while the subplots included three rates of naphthalene acetic acid (NAA) (a synthetic auxin) (0, 20, and 40 mg NAA L^–1^), which were prepared with distilled deionized water. After 5 days of flax budding, each plot was sprayed with a low-pressure hand-wand sprayer on the leaves, applying 50 mL m^–2^. Nitrogen fertilization was applied at a rate of 120 kg N ha^–1^ as urea, with 70% was applied as basal fertilization and the remaining 30% at the budding stage just before a significant rainfall occurred. Potassium sulfate was applied at 75 kg ha^–1^ for potassium fertilization, while calcium super-phosphate served as the basal fertilizer for P fertilization. No irrigation was provided to the crop. Manual weeding took place between sowing and harvesting. Flax was harvested by hand.

### Preplant soil sampling and analysis

Soils were collected from the upper 30 cm prior to sowing of the experiment and analyzed according to the methodology of Bao (2000). Specifically, pH was measured using potentiometry, soil organic matter content was determined by potassium dichromate volumetry, alkali-hydrolysable N was quantified using the alkali hydrolysis diffusion method, available potassium was obtained *via* flame photometry, and available P in soil was determined using the colorimetric Molybdenum-Blue method. The soil pH, organic matter, alkali-hydrolyzable N, available K, and available P at the experimental site were of 7.14 and 7.68, 12.8 and 13.4 g kg^–1^, 52.1 and 58.3 mg kg^–1^, 129.3 and112.6 mg kg^–1^, and 9.7 and 9.9 mg kg^–1^ in 2019 and 2020, respectively.

### Sampling and analysis

**Figure 1:**
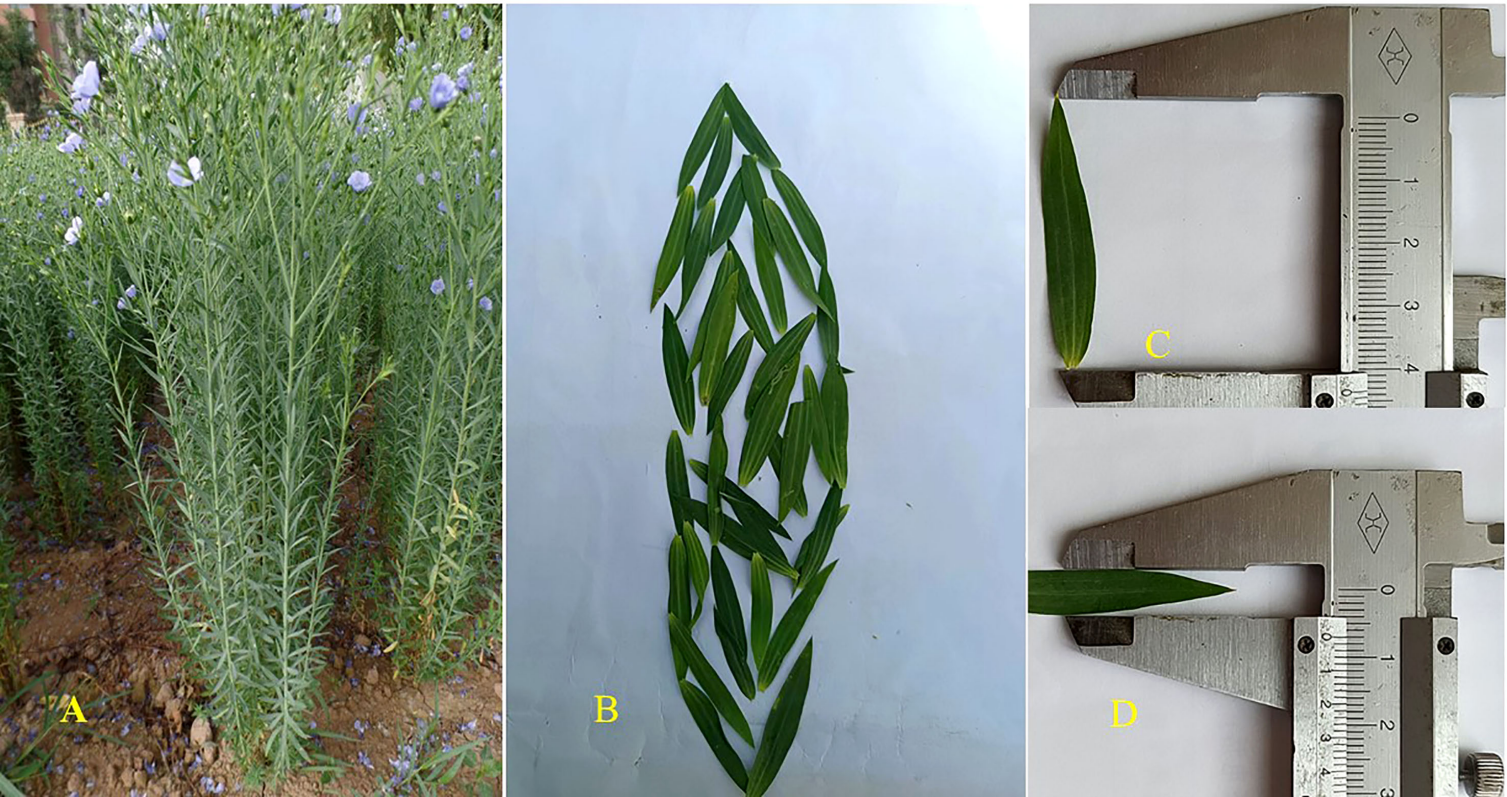
Morphology of plant at flowering **(A)**, leaf **(B)**, and the length of fully prolonged leaf **(C)** and width of fully prolonged leaf **(D)** of flax.

After 24 h of NAA spray on leaves during budding, 30 flax plants were chosen from the two central rows of each plot and separated into leaves and other parts (O'Neill et al., 2010; Xing et al., 2018; Cai et al., 2022). Once detached from the plants, leaf samples were frozen in liquid N at −80°C to measure the activities and relative expression levels of SPS and Rubisco. At flowering (approximately 7 days after initial flowering) (**Figure 1**), total aboveground dry matter, stem dry matter, leaf dry matter, and flower dry matter were determined. At maturity, the stem, leaves, non-seed reproductive structures (including peduncle, flower bud, sepal, carpopodium, and pericarp), and seeds were assessed. On the sampling date, a 1-m length of plant rows was randomly selected from the two central rows of each plot, recorded numbers of fruiting branches, tillers (the secondary basal stems of flax are referred to as tillers) and capsules per plant, as well as and seeds per capsule (Hocking and Pinkerton, 1993). Plant height was measured from the base to the highest bud and then separated into leaves, stems, non-seed reproductive structures, and seeds. The various vegetative organs were individually dried at 105°C for 2 hours followed by drying at 80°C until constant weight (Xie et al., 2014).

On the day of harvest, each plot’s crop was harvested separately using a sickle to determine its seed yield.

### Measurements

The N concentrations in the various organs were measured using micro-Kjeldahl method, as described by Lithourgidis et al. (2007). The P concentrations in the different plant organs were determined by the Colorimetric Molybdenum-Blue method (Lithourgidis et al., 2007). In the present study, the following formulae were computed according to the methods outlined by Dordas (2009) and de Oliveira Silva et al. (2020), as follow:


(1) $$Nitrogen\;translocation\;\left(kg\;ha^{-1}\right)\;=N\;accumulation\;at\;flowering\;-\;\left[leaf\;+\;stem\;+\;non-seed\right]\;N\;accumulation\;at\;maturity$$

(2) $$Phosphorus\;translocation\;\left(kg\;ha^{-1}\right)\;=P\;accumulation\;at\;flowering\;-\;\left[leaf\;+\;stem\;+\;non-seed\right]\;P\;accumulation\;at\;maturity$$

(3) Nitrogen harvest index (NHI) = N accumulation in seed/N accumulation at maturity

(4) Phosphorus harvest index (PHI) = P accumulation in seed/P accumulation at maturity

Additionally, the calculation of N and P assimilation post-flowering was determined using the following formula:


(5) $$Post-flowering\;N\;assimilation\;\left(kg\;ha^{-1}\right)\;=\;seed\;N\;accumulation\;atmaturity-N\;translocation$$

(6) $$Post-flowering\;P\;assimilation\;\left(kg\;ha^{-1}\right)\;=\;seed\;P\;accumulation\;atmaturity-P\;translocation$$

Activity of SPS was determined using the methods described by Mulyatama et al. (2022). In brief, the extract samples were incubated for 0, 5 and 10 min at 25°C and the reactions were terminated using 1 M NaOH. After addition of 0.25 mL resorcinol (1%) and 0.75 mL of 30% HCl, the sample was measured using spectrophotometer (U-5100 UV/VIS Hitachi HIgh-Tech Science Corporation Tokyo Japan) at 520 nm. Rubisco activity was measured following the protocol outlined by Wang et al. (2022). Leaves were homogenized in 9 mL pre-cooled (4°C) phosphate-buffered saline solution (pH 7.4). The resulting supernatant was collected after centrifugation at 5000 rpm for 25 min at 4°C. Rubisco activity was quantified using an enzyme-linked immunosorbent assay (ELISA) kit from Shanghai Guduo Biotechnology Co., Ltd., Shanghai, China, according to the manufacturer’s instructions. The absorbance of the sample was measured at a wavelength of 450 nm using a microplate reader (SpectraMax CMax Plus; Molecular Devices, San Jose, CA, USA).

Total RNA of leaves of flax samples as described above was extracted using the TransZol Up Plus RNA Kit (ER501-01, TransGen Biotech Co., Ltd.). The PrimeScript™ RT reagent Kit with gDNA Eraser (Perfect Real Time) (RR047A, Takara, Biotech Co., Ltd., Beijing, China) was used to reverse transcribe total RNA into cDNA and remove genomic DNA mixed in the cDNA, according to the manufacturer’s protocol. The reverse transcribed cDNAs were used for real-time quantitative PCR (RT-qPCR), which was performed on an Applied Biosystems Quant-Studio™ 5 platform (Thermo Fisher Scientific, Waltham, MA, USA). Four *LuSPS* genes and 1 *LuRBCL* gene were obtained from the genome database of *L. usitatissimum* (GenBank number: QMEG00000000) (Zhang et al., 2020). The primers were designed with the Primer premier 5.0 software and synthesized by TsingKe Biological Technology Co., Ltd. (Xi’an, China) (**Table S1**). *LuGADPH* was used for internal control (Huis et al., 2010; Zhang et al., 2020). Heiff^®^ qPCR SYBR^®^ Green Master Mix kit (Low Rox Plus) (Yeasen Biotech Co., Ltd.) was used for 20 μL PCR reactions as follow: 95°C for 30 s, and 40 cycles of 95°C for 5 s and 60°C for 34 s. Three independent bio-logical replicates were performed and triplicate technical quantitative assays were per-formed. The relative expression level (REL) of each sample was estimated according to the following equation as described by Livak and Schmittgen (2001): REL = 2^– ΔΔCt^, where the ΔΔCt value was the ΔCt value of the target gene in each sample minus the ΔCt value of the calibrator. The ΔCt value of the target gene came from the difference between the Ct value of the target gene and the Ct value of *LuGADPH* in each sample. The ΔCt value of the calibrator was the mean value from the difference between the Ct value of the target gene and the Ct value of *LuGADPH* in a sample under control conditions. The Ct value of the target gene and *LuGADPH* in samples was obtained from the Applied Biosystems Quant-Studio™ 5 platform.

### Data analysis

The data were subjected to analysis of variance (ANOVA) using SPSS (version 19, Inc., Chicago, IL, USA). Means were compared using the Tukey test with a significance level of 0.05. All RT-qPCR data were presented as means ± SE (n = 3).

## Results

### Phosphorus and NAA on the RT-q PCR of *LuSPS* and *LuRubisco*

**Figure 2:**
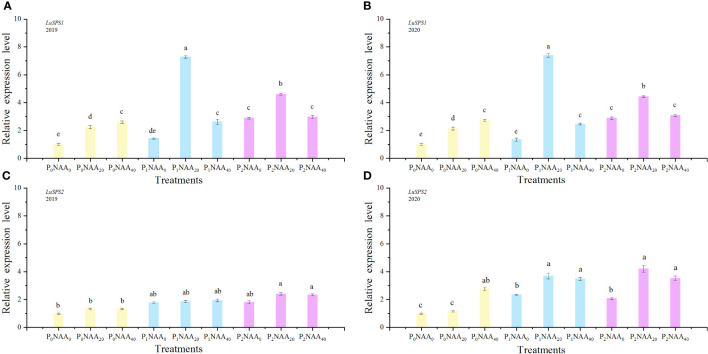
Effect of phosphorus and naphthalene acetic acid levels on the relative expression levels of *LuSPS1* **(A)** (2019) and **(B)** (2020) as well as *LuSPS2* **(C)** (2019) and **(D)** (2020). P stands for phosphorus; NAA refers to naphthalene acetic acid. P_0_, P_1_, and P_2_ represent 0, 67.5, and 135.0 kg P_2_O_5_ ha^−1^, respectively. NAA_0_, NAA_20_, and NAA_40_ represent 0, 20, and 40 mg NAA L^−1^, respectively. Different letters indicate means that are markedly different at *p*<0.05 based on the Tukey test.

**Figure 3:**
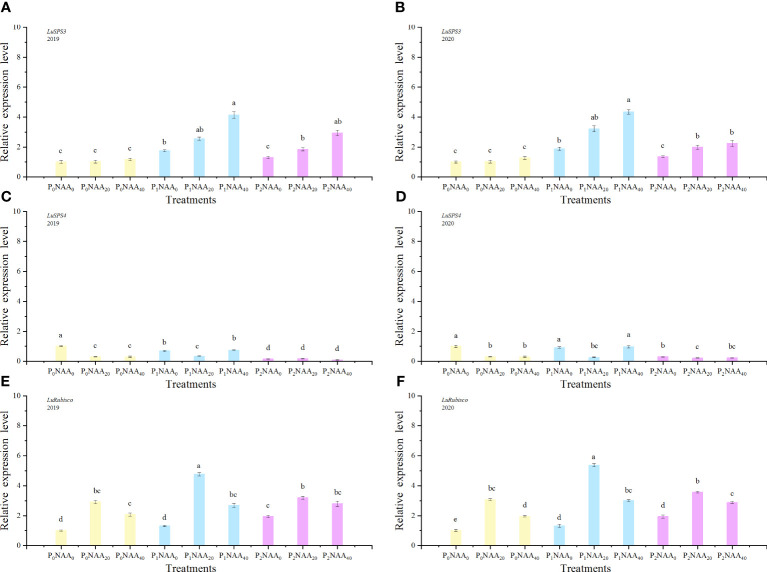
Effect of phosphorus and naphthalene acetic acid levels on the relative expression levels of *LuSPS3* **(A)** (2019) and **(B)** (2020), *LuSPS4* **(C)** (2019) and **(D)** (2020) as well as *LuRubisco* **(E)** (2019) and **(F)** (2020). NAA refers to naphthalene acetic acid. P_0_, P_1_, and P_2_ represent 0, 67.5, and 135.0 kg P_2_O_5_ ha^−1^, separately. NAA_0_, NAA_20_, and NAA_40_ represent 0, 20, and 40 mg NAA L^−1^, respectively. Different letters indicate significant differences at *p*<0.05 according to the Tukey test.

We examined the expression of *LuSPS1, LuSPS2, LuSPS3, LuSPS4*, and *LuRubisco* in flax leaves under various P and NAA treatments (**Figures 2**, **3**). The expression level of *LuSPS1* was significantly induced by P, NAA, and their interaction (**Table 1**). Specifically, P treatments increased the expression of *LuSPS1* by an average of 154% (in 2019) and 138% (in 2020), compared to no application of P. Meanwhile, NAA treatments led to a significant increase in the expression level of *LuSPS1* by an average of 111% across both years, as compared with no NAA treatments. The highest level of *LuSPS1* expression was achieved when application of 67.5 kg P_2_O_5_ ha^–1^ and 20 mg NAA L^–1^ in both years (**Figures 2A, B**), approximately 634% higher than that observed under control conditions (0 kg P_2_O_5_ ha^–1^ with 0 mg NAA L^–1^).

**Table 1:** Analysis of variance of various parameters that were measured in this study according to year, phosphorus and naphthalene acetic acid.

Parameters	Year (Y)	P	NAA	Y×P	Y×NAA	P×NAA	Y×P×NAA
Relative expression level of *LuSPS1*	ns	*	**	ns	ns	*	ns
Relative expression level of *LuSPS2*	ns	*	*	ns	*	ns	ns
Relative expression level of *LuSPS3*	ns	**	**	ns	ns	ns	ns
Relative expression level of *LuSPS4*	ns	*	*	*	*	*	ns
Relative expression level of *LuRubisco*	*	*	*	ns	ns	*	ns
Sucrose phosphate synthase (SPS) activity	*	*	*	ns	ns	**	ns
Ribulose-1,5-bisphosphate carboxylase/oxygenase (Rubisco) activity	*	**	**	ns	*	**	ns
Nitrogen accumulation at flowering	*	**	*	ns	ns	ns	ns
Nitrogen accumulation at maturity	**	**	**	ns	ns	*	ns
Nitrogen assimilation post-flowering	**	**	**	*	*	**	ns
Nitrogen translocation	ns	*	*	ns	ns	*	ns
Nitrogen harvest index	ns	**	**	ns	ns	**	ns
P accumulation at flowering	ns	**	*	ns	ns	ns	ns
P accumulation at maturity	*	**	*	ns	ns	*	ns
P assimilation post-flowering	ns	**	*	ns	ns	*	ns
P translocation	*	**	*	*	ns	*	ns
P harvest index	ns	*	**	ns	ns	**	ns
Plant height	*	ns	ns	*	ns	ns	*
Number of fruiting branches per plant	ns	**	*	ns	ns	*	*
Number of tillers per plant	*	*	**	ns	ns	**	ns
Seed yield	**	**	**	ns	ns	**	ns
Number of capsules per plant	*	**	**	ns	*	**	ns
Number seeds of per capsule	ns	ns	ns	*	ns	ns	ns
1000-seed weight	ns	*	ns	ns	ns	*	ns

P stands for phosphorus. NAA refers to naphthalene acetic acid. * and ** represent significance at the 0.05 and 0.01 level of probability, respectively. ns represent not significant.

Phosphorus significant influenced the expression of *LuSPS2*, with an increase of 93 and 69% in 2019 and 2020, respectively, compared to no P treatment. The expression of *LuSPS3* was also affected by both P and NAA treatments, resulting in increase of 128 and 129% with P treatments in both years, respectively, compared with no P, while NAA application led to respective increases of 72 and 52% in 2019 and 2020 compared to no spray NAA. The expression of *LuSPS4* significantly decreased with the application of NAA, exhibiting a decrease of 72 and 52% in 2019 and 2020, respectively, compared to no NAA treatment. Furthermore, the interaction between P and NAA as well as the year, P, and NAA interaction had an influenced on *LuSPS4* expression (**Table 1**). The highest level of *LuSPS4* expression was observed at 0 kg P_2_O_5_ ha^–1^ with 0 mg NAA L^–1^ in two years. The lowest value of *LuSPS4* expression level was observed at 135.0 P_2_O_5_ ha^–1^ with 40 mg NAA L^–1^ in 2019 (0.09) and at 135.0 P_2_O_5_ ha^–1^ with 20 mg NAA L^–1^ in 2020 (0.22) (**Figures 3C, D**).

The expression level of *LuRubisco* was influenced by year, P, NAA, and the between P and NAA interaction (**Table 1**). As the P rate increased, there was a corresponding increase in the expression level of *LuRubisco*. Compared to the zero P control, fertilized flax showed an increase of 41 and 73% in *LuRubisco* expression level when averaged over 67.5 and 135.0 kg P_2_O_5_ ha^–1^, respectively. The expression level of *LuRubisco* initially increased but then decreased with increasing NAA rate. The maximal expression values of *LuRubisco* were 2.2 (in 2019) and 3.0 (in 2020) across three P rates, with the highest expression level being 4.8 (in 2019) and 5.4 (in 2020) at a rate of 67.5 kg P_2_O_5_ ha^–1^ with 20 mg NAA L^–1^(**Figures 3E, F**). Notably, the expression levels in NAA treatments exceeded those in zero NAA treatments by up to 114% in 2019 and 88% in 2020.

### Phosphorus and NAA on SPS and Rubisco activity

**Figure 4:**
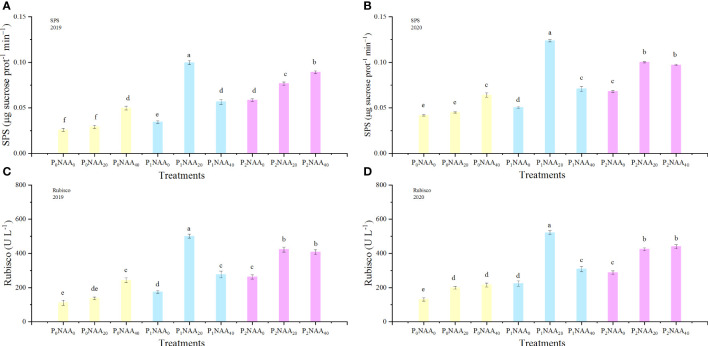
Effect of phosphorus and naphthalene acetic acid levels on the SPS **(A)** (2019) and **(B)** (2020) as well as Rubisco **(C)** (2019) and **(D)** (2020) activities. P refers to phosphorus; NAA stands for naphthalene acetic acid. P_0_, P_1_, and P_2_ represent 0, 67.5, and 135.0 kg P_2_O_5_ ha^−1^, respectively. NAA_0_, NAA_20_, and NAA_40_ represent 0, 20, and 40 mg NAA L^−1^, respectively. Different letters indicate significant differences at *p*<0.05 based on the Tukey test.

The activity of SPS was affected by year, P, NAA, and the interaction between P and NAA (**Table 1**). Compared to zero P, SPS activity increased by 118 and 71% with P treatments in 2019 and 2020, respectively. Additionally, the application of NAA resulted in an average increase of SPS activity by 65 and 55% in 2019 and 2020, respectively, compared with zero NAA. The maximum value of SPS activity was achieved with the application of 67.5 kg P_2_O_5_ ha^–1^ with 20 mg NAA L^–1^; however, the lowest level of SPS activity was observed at 0 kg P_2_O_5_ ha^–1^ with 0 mg NAA L^–1^ (**Figures 4A, B**).

The activity of Rubisco in flax leaves was affected by P (**Table 1**). Consistent with the trend of *LuRubisco* expression level, Rubisco activity increased as P increased. Compared to zero P, an average increase in Rubisco activity was 108% (in 2019) and 102% (in 2020) in the P treatments. Additionally, NAA application had an impact on Rubisco activity. The trend of Rubisco activity change was consistent with the response of *LuRubisco* expression level to NAA. Compared to zero NAA treatments, there was an average increase in Rubisco activity of 81% in 2019 and 64% in 2020. The peak Rubisco activity was obtained at 67.5 kg P_2_O_5_ ha^–1^ combined with 20 mg NAA L^–1^; however, the lowest value was observed under conditions of zero P and NAA conditions (**Figures 4C, D**).

### Phosphorus and NAA on N accumulation and translocation

Nitrogen accumulation in aboveground plant parts at flowering and maturity, as well as post-flowering N assimilation significantly increased by the application of P fertilizers in 2019 and 2020 years. Compared to zero P, the application of P fertilization resulted in an average increase of 12% in 2019 and 16% in 2020 in N accumulation at flowering. Furthermore, N accumulation at maturity showed a significant increase of 24% (in 2019) and 30% (in 2020) in P treatments compared with zero P. Moreover, N assimilation post-flowering improved by 51 and 59% in the P treatments in 2019 and 2020, respectively, compared to zero P. Additionally, the application of P resulted in an average increase of 15% in N translocation compared to without P treatment. As shown in **Table 1**, P had an influence on NHI. In this study, the NHI increased by 11 and 14% with the P treatments in 2019 and 2020, respectively.

The application of NAA had a significant effect on N accumulation at flowering and maturity, post-flowering N assimilation, and N translocation. Compared to the zero NAA treatment, the use of NAA increased N accumulation at flowering and maturity, N assimilation post-flowering, N translocation, and NHI by 11, 17, 28, 10, and 5% in 2019, respectively. In addition, compared to zero NAA, there was a significant increase in N accumulation at flowering and maturity, N assimilation post-flowering, N translocation, and NHI, by 12, 18, 30, 16, and 6% in the year of 2020 with the application of NAA.

The interaction between P and NAA had a significant effect on N accumulation at maturity, post-flowering N assimilation, N translocation, and NHI (**Table 1**). In both years, the maximum values of those indexes were observed when applying 67.5 kg P_2_O_5_ ha^–1^ combined with 20 mg NAA L^–1^; conversely, the lowest values were recorded under no application of P or NAA (**Table 2**). Compared with the lowest values, the maximum of these indexes increased by an average of 54, 50, 105, and 22%, respectively.

**Table 2:** Effect of phosphorus and naphthalene acetic acid levels on nitrogen accumulation, translocation, nitrogen assimilation post-flowering, and nitrogen harvest index of flax in 2019 and 2020 years at Dingxi, China.

Year	P rate	NAA rate	Nitrogen accumulation at flowering (kg ha^−1^)	Nitrogen accumulation at maturity (kg ha^−1^)	Nitrogen assimilation post-flowering (kg ha^−1^)	Nitrogen translocation (kg ha^−1^)	Nitrogen harvest index
2019	P_0_	NAA_0_	60.36b^†^	84.30g	23.94f	12.06d	0.43c
		NAA_20_	65.94b	91.33f	25.39f	13.70c	0.43c
		NAA_40_	66.83b	100.73e	33.90e	14.25b	0.48b
	P_1_	NAA_0_	63.89b	96.90e	33.00e	13.50c	0.48b
		NAA_20_	77.96a	126.96a	49.01a	15.75a	0.51a
		NAA_40_	72.15a	116.52c	44.37b	14.24b	0.50a
	P_2_	NAA_0_	69.38ab	106.60d	37.22d	13.84c	0.48b
		NAA_20_	74.73a	121.19b	46.46b	14.38b	0.50a
		NAA_40_	73.26a	115.03c	41.77c	14.74b	0.49b
2020	P_0_	NAA_0_	60.17b	88.37e	27.02e	10.04d	0.43d
		NAA_20_	65.41b	92.43e	28.20e	13.91c	0.44d
		NAA_40_	68.48b	105.17d	36.69d	13.79c	0.48c
	P_1_	NAA_0_	67.80b	103.19d	35.39d	15.07b	0.49c
		NAA_20_	81.66a	139.78a	58.12a	17.50a	0.54a
		NAA_40_	76.03a	127.35bc	51.32b	15.28b	0.52b
	P_2_	NAA_0_	70.83b	113.96c	43.13c	14.18c	0.50c
		NAA_20_	78.37a	130.54b	52.17b	15.71b	0.52b
		NAA_40_	75.32a	126.49bc	51.17b	14.86b	0.52b

P stands for phosphorus. NAA refers to naphthalene acetic acid. P_0_, P_1_, and P_2_ represent 0, 67.5 and 135.0 kg P_2_O_5_ ha^−1^, respectively. NAA_0_, NAA_20_, and NAA_40_ represent 0, 20, and 40 g l^−1^, respectively. † Means in the same column followed by the same letter do not differ significantly according to the Tukey test (*p* = 0.05).

### Phosphorus and NAA on P accumulation and translocation

Phosphorus accumulation at flowering, maturity, and assimilation post-flowering, as well as P translocation in aboveground plant parts and PHI increased significantly with the application of P fertilizers in both years. The P accumulation at flowering, maturity, and assimilation post-flowering, P translocation aboveground plant parts of flax as well as PHI were 113, 106, 88, 258, and 15% greater compared to the zero P application in 2019, and were 108, 99, 76, 274, and 14% greater in 2020.

The application of NAA significantly increased P accumulation at flowering, maturity, and assimilation post-flowering, as well as P translocation in aboveground plant parts and PHI in both years. Similarly, the P accumulation at flowering, maturity, and assimilation post-flowering, P translocation as well as PHI were 16, 16, 16, 29, and 6% greater compared to the zero NAA application in 2019, and were 17, 23, 46, 16, and 5% greater in 2020.

The interaction between P and NAA significantly affected P accumulation at maturity, assimilation post-flowering, P translocation, and PHI (**Table 1**). The peak of P accumulation at maturity, assimilation post-flowering, P translocation, and PHI were obtained at 67.5 kg P_2_O_5_ ha^–1^ combined with 20 mg NAA L^–1^ in both years, while the lowest were observed when application 0 kg P_2_O_5_ ha^–1^ and 0 mg NAA L^–1^ in both years (**Table 3**). On average, the maximum of these indexes were 143, 117, 557, and 29% greater compared to the lowest in 2019, and were 176, 183, 394, and 25% greater in 2020.

**Table 3:** Effect of phosphorus and naphthalene acetic acid levels on phosphorus accumulation, translocation, phosphorus assimilation post-flowering, and phosphorus harvest index of flax in 2019 and 2020 years at Dingxi, China.

Year	P rate	NAA rate	P accumulation at flowering (kg ha^−1^)	P accumulation at maturity (kg ha^−1^)	P assimilation post-flowering (kg ha^−1^)	P translocation (kg ha^−1^)	P harvest index
2019	P_0_	NAA_0_	6.21d^†^	8.63e	2.42c	0.63d	0.35e
		NAA_20_	6.68c	9.28d	2.60c	0.93d	0.38d
		NAA_40_	7.52c	9.97d	2.45c	1.34c	0.38d
	P_1_	NAA_0_	12.52b	16.32c	3.80b	2.96b	0.41c
		NAA_20_	15.70a	20.97a	5.26a	4.14a	0.45a
		NAA_40_	14.46a	18.90b	4.44b	3.47a	0.42b
	P_2_	NAA_0_	13.51b	17.96b	4.46b	3.05b	0.42b
		NAA_20_	15.37a	20.53a	5.17a	3.54a	0.42b
		NAA_40_	15.28a	20.18a	4.89ab	3.66a	0.42b
2020	P_0_	NAA_0_	6.22d	8.37e	2.15e	0.89d	0.36d
		NAA_20_	7.85c	10.84d	2.99e	1.06c	0.37d
		NAA_40_	8.22c	11.52d	3.30d	1.17c	0.39c
	P_1_	NAA_0_	13.56b	17.03c	3.47d	3.67b	0.42b
		NAA_20_	16.99a	23.06a	6.08a	4.40a	0.45a
		NAA_40_	15.26a	21.01b	5.75a	3.54b	0.44a
	P_2_	NAA_0_	14.66b	18.80c	4.14c	3.43b	0.40c
		NAA_20_	16.30a	21.54b	5.24ab	4.28a	0.44a
		NAA_40_	15.86a	20.87b	5.01b	4.01a	0.43a

P stands for phosphorus. NAA refers to naphthalene acetic acid. P_0_, P_1_, and P_2_ represent 0, 67.5 and 135.0 kg P_2_O_5_ ha^−1^, respectively. NAA_0_, NAA_20_, and NAA_40_ represent 0, 20, and 40 g l^−1^, respectively. † Means in the same column followed by the same letter do not differ significantly according to the Tukey test (*p* = 0.05).

### Phosphorus and NAA on the growth phenotype of flax

In the present study, there was no significant difference in flax plant height among P, NAA, and their interaction (**Table 1**). However, the interaction among the year, P, and NAA affected plant height. The numbers of fruiting branches and tillers per plant were affected by P, NAA, and their interaction as shown in **Table 1**. The application of P fertilizer resulted in a significant increase in the number of fruiting branches per plant, with an average improvement of 15 and 17% observed in 2019 and 2020, respectively, compared to plants that did not receive any P treatment. Similarly, the use of sprayed NAA led to an average increase of 11% (in 2019) and 10% (in 2020) in the number of fruiting branches per plant when compared to plants that were not treated with NAA spray. The number of fruiting branches per plant was impacted by the year, P, and NAA interaction (**Table 1**). The highest number of fruiting branches per plant was recorded at 67.5 kg P_2_O_5_ ha^–1^ and 40 mg NAA L^–1^ in 2019, and at 67.5 kg P_2_O_5_ ha^–1^ and 20 mg NAA L^–1^ in 2020 (**Table 4**).

**Table 4:** Effect of phosphorus and naphthalene acetic acid levels on plant height, number of fruiting branches per plant, and number of tillers per plant of flax in 2019 and 2020 years at Dingxi, China.

Year	P rate	NAA rate	Plant height (cm)	Number of fruiting branches per plant	Number of tillers per plant
2019	P_0_	NAA_0_	71.70b^†^	18.13d	1.46d
		NAA_20_	75.86a	19.17c	2.04c
		NAA_40_	72.68ab	19.96c	2.20c
	P_1_	NAA_0_	69.54b	20.85b	2.21c
		NAA_20_	60.46c	22.87a	2.86b
		NAA_40_	70.22b	23.68a	3.23b
	P_2_	NAA_0_	68.28b	19.52c	3.15b
		NAA_20_	77.92a	21.85b	3.73a
		NAA_40_	78.00a	22.85a	4.02a
2020	P_0_	NAA_0_	75.26b	19.47d	1.80d
		NAA_20_	81.22a	20.01d	2.53c
		NAA_40_	85.26a	21.12c	2.58c
	P_1_	NAA_0_	78.62ab	22.76b	2.84c
		NAA_20_	76.76b	25.91a	3.36b
		NAA_40_	67.50c	25.23a	3.91a
	P_2_	NAA_0_	64.52d	20.91c	3.01b
		NAA_20_	70.18c	23.78b	3.83a
		NAA_40_	75.54b	23.41b	4.47a

P stands for phosphorus. NAA refers to naphthalene acetic acid. P_0_, P_1_, and P_2_ represent 0, 67.5 and 135.0 kg P_2_O_5_ ha^−1^, respectively. NAA_0_, NAA_20_, and NAA_40_ represent 0, 20, and 40 g l^−1^, respectively. † Means in the same column followed by the same letter do not differ significantly according to the Tukey test (*p* = 0.05).

The number of tillers per plant increased with increasing P supply. Compared to no application of P, the use P treatments resulted in an average increase of 68% in 2019 and 55% in 2020. Furthermore, the application of NAA led to an average increase in the number of tillers per plant by 33 and 35% in 2019 and 2020, respectively, compared to zero NAA. Additionally, the highest values were observed at a combination of 135 kg P_2_O_5_ ha^–1^ and 40 mg NAA L^–1^ in both years (**Table 4**).

### Phosphorus and NAA on seed yield and yield components

Phosphorus significantly impacted the seed yield of flax, with an increase in seed yield as P supply rate increased. Notably, no difference was found between 67.5 and 135.0 kg P_2_O_5_ ha^–1^. The application of P fertilizer resulted in an average increment in seed yield of 12% (2019) and 14% (2020) when compared to zero P. Furthermore, both capsules per plant and 1000-seed weight were influenced by P fertilizer (**Table 1**). Capsules per plant showed an average improvement of 6 and 9% in the P treatments in 2019 and 2020, respectively, compared to no application of P. Additionally, the addition of P resulted in a 3% increase (in both years) in 1000-seed weight when compared to no application of P.

Seed yield was significantly impacted by the application of NAA. Compared to the zero NAA treatment, the application of NAA led to an average increase in seed yield of 8% in 2019 and 9% in 2020, respectively. Furthermore, the application of NAA significantly affected the capsules per plant of flax, with an average increase of 12%, when compared to the zero NAA treatment. Moreover, the interaction between P and NAA had a significant influence on the seed yield, capsules per plant, and 1000-seed weight of flax (**Table 1**). The highest seed yield of 1891 kg ha^–1^ in 2019 and 2029 kg ha^–1^ in 2020 was achieved with an application of 67.5 kg P_2_O_5_ ha^–1^ and 20 mg NAA L^–1^ (**Table 5**). On average, the maximum seed yield was 36% greater than the lowest value in both years. Additionally, the maximum capsules per plant were observed at 67.5 kg P_2_O_5_ ha^–1^ and 20 mg NAA L^–1^ in both 2019 and 2020; whereas the highest 1000-seed weight was observed at a rate of 135.0 kg P_2_O_5_ ha^–1^ and 40 mg NAA L^–1^. On average, the maximum values increased by an average of 3% compared with the lowest 1000-seed weight (**Table 5**).

**Table 5:** Effect of phosphorus and naphthalene acetic acid levels on seed yield and yield components of flax in 2019 and 2020 years at Dingxi, China.

Year	P rate	NAA rate	Seed yield (kg ha^−1^)	Number of capsules per plant	Number of seeds per capsule	1000-seed weight
2019	P_0_	NAA_0_	1396.11e^†^	16.67d	6.82a	6.01c
		NAA_20_	1511.66d	18.23c	6.91a	6.06bc
		NAA_40_	1485.00d	19.60b	7.12a	6.12b
	P_1_	NAA_0_	1487.34d	17.84c	7.05a	6.12b
		NAA_20_	1890.67a	21.26a	7.12a	6.13b
		NAA_40_	1491.34d	20.22b	7.20a	6.24b
	P_2_	NAA_0_	1633.35c	17.86c	7.12a	6.31a
		NAA_20_	1700.33b	19.78b	7.05a	6.35a
		NAA_40_	1677.00bc	18.09c	7.31a	6.43a
2020	P_0_	NAA_0_	1480.67e	15.41e	6.86a	6.05c
		NAA_20_	1789.33d	20.46c	7.09a	6.12c
		NAA_40_	1786.34d	22.04b	7.05a	6.14c
	P_1_	NAA_0_	1837.66c	19.88d	7.15a	6.12c
		NAA_20_	2028.67a	23.47a	7.13a	6.33b
		NAA_40_	1886.69b	20.92c	7.22a	6.45a
	P_2_	NAA_0_	1904.34b	20.50c	6.94a	6.27b
		NAA_20_	1981.01a	21.21bc	6.99a	6.32b
		NAA_40_	1909.03b	20.82c	7.05a	6.51a

P stands for phosphorus. NAA refers to naphthalene acetic acid. NAA refers to naphthalene acetic acid. P_0_, P_1_, and P_2_ represent 0, 67.5 and 135.0 kg P_2_O_5_ ha^−1^, respectively. NAA_0_, NAA_20_, and NAA_40_ represent 0, 20, and 40 g l^−1^, respectively. † Means in the same column followed by the same letter do not differ significantly according to the Tukey test (*p* = 0.05).

## Discussion

### Effect of P

Our research indicated the application of P fertilizer enhanced SPS activity in flax by up-regulating the relative expression level of *LuSPS1-3* while down-regulating that of *LuSPS4*. In addition, this study has also revealed that the relative expression level of *LuRubisco* in flax leaves significantly increased under P fertilization treatments. Furthermore, we observed a similar trend between Rubisco activity and the relative expression level of *LuRubisco* in response to P application. In a study of wheat, the function of SPS was initially discovered by Leloir and Cardini (1955). Subsequently, many studies have demonstrated that SPS is the pivotal enzyme in sucrose biosynthesis, which is associated with crop growth and yield (Castleden et al., 2004). Recent research has shown that P fertilizer up-regulates the activity of SPS in citrus fruit (Wu et al., 2021). This finding aligns with our study results, which demonstrate P fertilizer increased flax leaves SPS activity. In cotton, the addition of P has been shown to enhance SPS activity in cottonseed kernel (Wang et al., 2023). Moreover, numerous studies have revealed that plant species possess multiple *SPS* genes and their expression varies depending on developmental stages, tissue types and environmental cues (Lutfiyya et al., 2007; Ma et al., 2020). Obviously, the application of P fertilizer can promote photosynthetic C metabolism, as judged from the relative expression levels of *LuSPS1-3* and *LuRubisco*, as well as SPS and Rubisco activities. This was related to higher photosynthesis efficiency. Li et al. (2022) demonstrated that Pi deficiency regulated photosynthesis-related genes at the transcriptional level, thereby inhibiting photosynthesis. Moreover, Kayoumu et al. (2023) reported that appropriate P enhanced the photosynthesis of cotton.

Previous study found that P treatments significantly increased N and P accumulations at flowering and maturity as well as post-flowering N and P assimilations and translocations on durum wheat (Dordas, 2009). These findings were in agreement with our current results. In addition, Xie et al. (2023) have found that the P fertilized flax enhanced the N uptake in aboveground parts. Those results showed that P enhanced N transport, reduction and assimilation. At the meantime, Dordas (2009) also reported that the application of P did not affect the NHI and PHI of durum wheat, this result is at odds with our studies. These differences were probably attributable to species, soil types, and sink capacity.

To meet the demand for food from the fast-growing global population, crop production will need to increase by approximately 60% (Muhie, 2022). The flexible management practice of P fertilization can enhance crop productivity. Application of P treatments significantly increased the numbers of fruiting branches and tillers per plant. In this respect, our results agreed with those of Hocking and Pinkerton (1993). Moreover, this study found that P application improved flax seed yield, which is consistent with previous reports (Muhammad et al., 2020; Xie et al., 2020; Xie et al., 2022). Similar results have been observed in other species, such as soybean (Yin et al., 2016; Taliman et al., 2019), pea, canola (Yin et al., 2016), wheat (Škarpa et al., 2021), maize (Škarpa et al., 2021), and rice (Škarpa et al., 2021). In this study, the application of P fertilizer had a significant influence on both 1000-seed weight and the number of capsules per plant in flax, which is consistent with previous research findings (Hocking and Pinkerton, 1993; Xie et al., 2016). In the present study, the increase in seed yield due to P fertilization may be a result of improvement fruiting branches per plant, capsule per plant and the 1000-seed weight. The rise in seed yield with fertilizer P application is likely linked to enhance C and N assimilation (Lawlor, 2002; Ren et al., 2020) and photosynthesis efficiency (Li et al., 2022).

### Effect of NAA

Previous studies have demonstrated that exogenous auxin can stimulate root system growth in plants, thus improving their ability to absorb nutrient and metal ion (Ji et al., 2015; Zaid et al., 2020; Chen et al., 2021). However, there has been limited research on the effects of application exogenous NAA to crops for increasing N uptake. In the current study, spray of NAA resulted in an increase in N and P accumulation at flowering and maturity, N and P assimilation post-flowering, N and P translocation, and NHI and PHI. Previous research has reported that exogenous application of IAA significantly promoted uranium (U) and cadmium (Cd) translocation from roots to shoots in sunflowers (Chen et al., 2021).

In this study, the application of NAA resulted in a significant increase in seed yield of flax. Previous studies on alfalfa and faba bean have also reported similar results, with a 23% increase in seed yield observed after NAA application (Zhang et al., 2009). Additionally, research on rice has identified that auxin can improve grain yield (Aryan et al., 2023). In our study, we found that NAA increased the number of fruiting branches per plant, tillers per plant, and capsules per plant and 1000-seed weight. The reasons for this may be adequate C and N assimilation after NAA application. In another study, Mousavi et al. (2022) observed that foliar application of IAA had a significantly positive effect on seed yield and yield components in safflower. However, it should be noted that Giannakoula et al. (2012) reported no significant effect of NAA on 1000-seed weight in lentil. The inconsistency among various studies may be attributable to differences in genotypes, type of auxin, and climate conditions. Additionally, the application of NAA increased the number of capsules per plant and seed yield. This can be attributed to NAA enhancing C and N metabolism in plants by up-regulating the SPS and Rubisco activities as well as N absorption, translocation, and assimilation, which promote photosynthesis resulting in seed production as well as flowering and seed formation. Further research is required to validate the mechanism for increasing seed yield through P and NAA application.

This is the first report demonstrating that the supply of P and NAA affects the relative expression of *LuSPS1, LuSPS2, LuSPS3*, *LuSPS4*, and *LuRubisco* as well as SPS and Rubisco activities, N and P accumulation, assimilation, and translocation in flax.

### Effect of the interaction

In this study, the interaction between NAA and P led to increasing SPS and Rubisco activities as well as N translocation and assimilation probably due to coordinating regulation of C and N assimilation (Lawlor, 2002; Ren et al., 2020), maintain a higher relatively stable C and N metabolism (Ren et al., 2020), and sustain C/N balance (Gong et al., 2020; Liu et al., 2022), and improve photosynthesis (Li et al., 2022). As a result, the P and NAA interaction increased significantly seed yield of flax, probably due to (i) with sufficient NO_3_^−^ and CO_2_ assimilation, the supply of assimilates from C and N assimilation to developing meristems is adequate to maintain their growth, resulting in an increase in tillers, fruiting branches, capsules per plant, and seed production; and (ii) the capacity of seeds to grow is enhanced, possibly partly explained by increase in cells with greater enzyme capacity. Given adequate assimilates during seed filling, more seeds are filled and they are larger. These factors collectively contribute to large yield (Lawlor, 2002). Furthermore, the complex mechanism of involved in others enzymes activities on C and N metabolites of flax with the combination of P and NAA for increasing seed yield need further to explore in the future.

Moreover, the interaction between year and P affected the relative expression level of *LuSPS4*, plant height, and seeds of per capsule of flax. The year and NAA interaction influenced Rubisco activity, and capsules per plan. Moreover, the interaction among the year, P, and NAA impacted plant height and number of fruiting branches per plant of flax.

## Conclusion

Effective management practices are critical for the economic viability of crop production and environmental sustainability. To estimate the effect of P and NAA rates on seed yield, we first elaborate on the response of *LuSPS1, LuSPS2, LuSPS3*, *LuSPS4*, and *LuRubisco* genes’ relative expression levels as well as SPS and Rubisco activities, N and P accumulation and translocation in flax under different P and NAA rates. Our study reveals that application of P and NAA significantly increased the relative expression levels of *LuSPS1*, *LuSPS3*, and *LuRubisco* genes, SPS and Rubisco activities, N and P accumulation at flowering and maturity, post-flowering N and P assimilation, N and P translocation, NHI, fruiting branches per plant, tillers per plant, capsules per plant, 1000-seed weight, and seed yield of flax.

**Figure 5:**
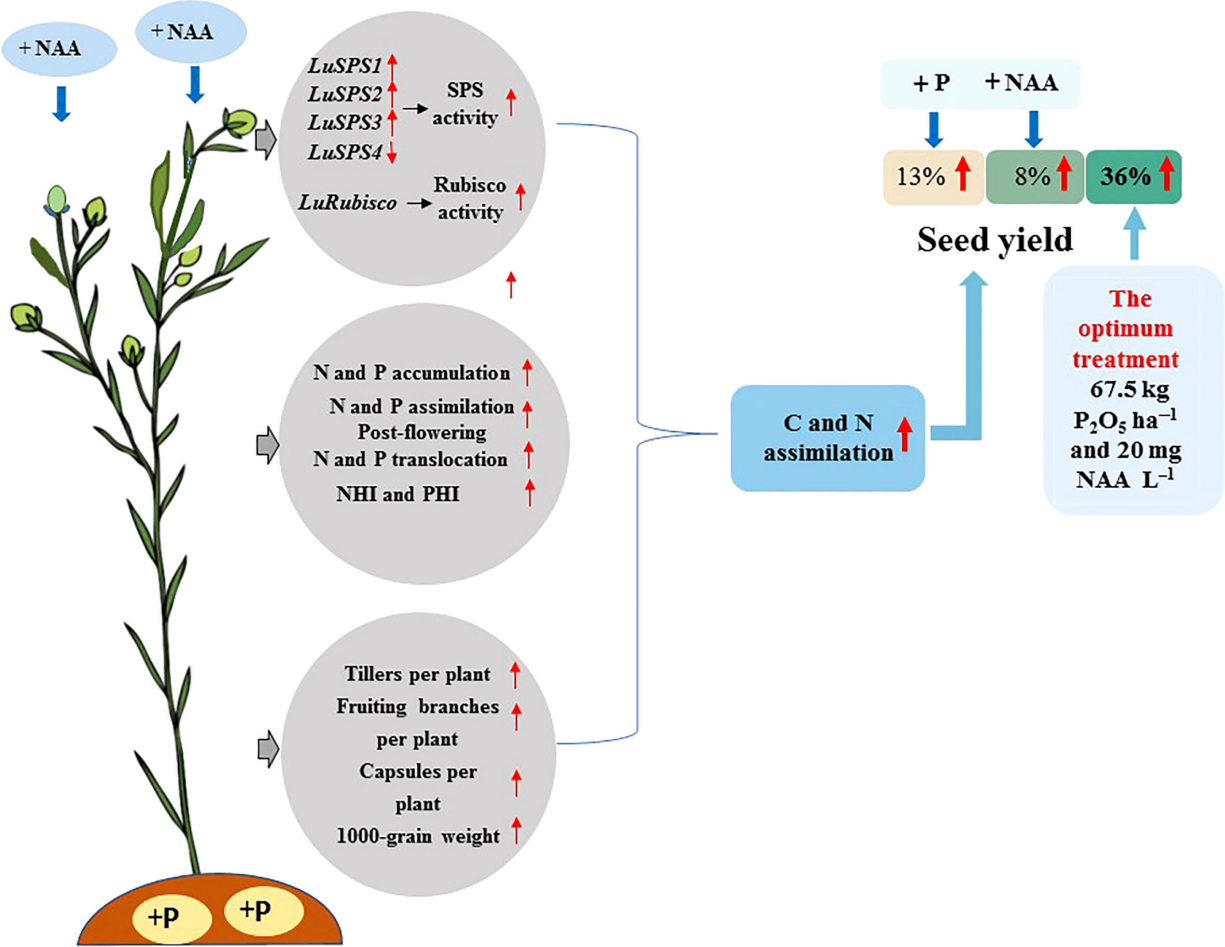
Appropriate combination of P and NAA increase seed yield of flax by maintaining higher C and N assimilation.

The highest seed yield (1891 kg ha^−1^ in 2019 and 2029 kg ha^−1^ in 2020) were recorded with the application of 67.5 kg P_2_O_5_ ha^–1^ and 20 mg NAA L^–1^. It appears that an appropriate combination of P and NAA can be applied in flax production to increase seed yield by maintaining higher C and N assimilation under rain-fed conditions as shown in **Figure 5**.

## Data availability statement

The original contributions presented in the study are included in the article/**Supplementary Material**. Further inquiries can be directed to the corresponding author.

## Author contributions

YX analyzed the data and prepared the first draft. HD and LW as project administration. JZ conceived the conceptualization and methodology for the experiments. ZD, WJL, and YQ investigated the manuscript. WZ, KD, XRW, YJZ, YCZ, XZW, WL, and LRZ helped in experiments and data collection. All authors contributed to the article and approved the submitted version.

## Publisher’s notes

All claims expressed in this article are solely those of the authors and do not necessarily represent those of their affiliated organizations, or those of the publisher, the editors and the reviewers. Any product that may be evaluated in this article, or claim that may be made by its manufacturer, is not guaranteed or endorsed by the publisher.
