# Supplementary material for: Phosphorus and naphthalene acetic acid increased the seed yield by regulating carbon and nitrogen assimilation of flax
Source: Front Plant Sci. 2023 Aug 30;14:1228755. doi: 10.3389/fpls.2023.1228755 (PMC10499554; doi:10.3389/fpls.2023.1228755)
Supplement: Supplementary file 1 [file Table_1.docx]

**Supplementary Table S1** Primer sequences for RT-qPCR analysis.

| **Gene** | **Gene ID** | **Forward primer** | **Reverse primer** | **Product length (bp)** |
| --- | --- | --- | --- | --- |
| *LuSPS1* | L.us.o.m.scaffold4.510 | GATGGGGTTGTGAAGGTCTAAAA | CTGGATGGGGCTTGACTGC | 101 |
| *LuSPS2* | L.us.o.m.scaffold149.32 | GTTGACTTCGGGTGTTGAGGAT | TCGTGGACCAAACGGGATT | 88 |
| *LuSPS3* | L.us.o.m.scaffold0.256 | CAACAACAAGGGCGGAGCG | ATTCTTTGGCAGATTCAGGAGG | 120 |
| *LuSPS4* | L.us.o.m.scaffold229.1+A138:B1803 | TTGTCATTGCGGTTGATTGTG | CCCTGGACGCTTTCCTTTTC | 91 |
| *LuRBCL* | L.us.o.m.scaffold178.9 | GCGGTGGTATTTATTTCACTCAAG | CGTCAAATTGTAATACGGAATCATCT | 135 |
| *LuGADPH* | CV478202 | CTTTACCCTCAGCAAATCCG | AGGTTCTTCCCGCTCTCAAT | 138 |
